# Hospital and Institutional News

**Published:** 1917-06-23

**Authors:** 


### HOSPITAL AND INSTITUTIONAL NEWS.

### THE LATE LIEUTENANT MACKINTOSH.

THE sympathy of the whole hospital world will go out to Colonel Donald Mackintosh, C.B., of the Western Infirmary, Glasgow, and Mrs. Mackintosh, whose only son Lieutenant Donald Mackintosh, late Seaforth Highlanders, after being wounded in April last year, and having been reported wounded and missing for some time, is now known to have been killed in action on April 11, 1917. Lieut. Mackintosh was only twenty-one years of age. His Majesty the King has been pleased to approve the award of the Victoria Cross to Lieut. Donald Mackintosh for "gallantry beyond all praise" and for most conspicuous bravery and resolution in the face of intense machine-gun fire. During the initial advance he was shot through the right leg, but though crippled he continued to lead his men and captured the trench. In the captured trench Lieut. Mackintosh collected men of another company who had lost their leader and drove back a counter-attack. He was again wounded, and although unable to stand he continued nevertheless to control the situation. With only fifteen men left, he ordered his party to be ready to advance to the final objective, and with great difficulty got out of the trench and encouraged his men to advance. He was again wounded and fell. The gallantry and devotion to duty of this officer were beyond all praise. Lieut. Mackintosh died for his country, and his noble conduct and example will prove an inspiration to the younger men of our race, and we hope, too, an abiding comfort and pride to his parents and friends.

### THE AIR RAID ON LONDON.

THE aid raid on London, which commenced about 11 A.M. on Wednesday, the 13th instant, differed from all the raids experienced in Zeppelin days from the fact that in the case of Zeppelins all hospitals received "a first warning" of every expected raid and subsequently "an urgent warning," when all stood at their places until the "All clear" signal followed. In the latest London raid the hospitals had practically no warning. This was the case at the London, Poplar, and Metropolitan Hospitals, while at St. Bartholomew's Hospital a few minutes' notice only was received. We understand that the difficulty lies in giving the hospitals early notice when an air raid takes place in the daytime, and the attacking fleet consists of fast-flying aeroplanes. There are aeroplanes of this type which, if unimpeded, may arrive over London within half an hour of their appearance on the coast. Still, during fine and warm weather, especially, it is of the first importance that every great hospital shall have the amplest possible warning of a coming raid. This applies especially to hospitals with garden space, which are provided with bed balconies in ample proportion to the number of their in-patients.

### HOW TO WARN LONDON HOSPITALS.

WE have not space this week exhaustively to treat this matter, but a brief account of the actual experience of the authorities at the London Hospital on the 13th instant graphically exhibits the position of affairs. "The garden at the back of the hospital was on that day, at the time of the raid, very full of convalescent patients, who were out in wheeled-chairs, on stretcher beds, and a great number of them on couches, whilst several were walking about. All these patients had to be brought in by volunteers while the raid was in actual progress and while the raiders could be seen overhead pretty close to the hospital. The balconies also were crowded with beds wheeled out upon them. In any raid a set of routine things has to be done, and these take time—the visiting staff must be 'phoned and told what may be happening; housemen must be informed and got to their wards; engineers must get to their posts; operating theatres have to be got ready, etc. It will be readily seen that every minute of warning is of value to the hospital authorities and its patients. Within an hour of the raid the London Hospital had treated about 207 patients, admitting nearly 100 of them. Up to the night of the 19th inst. forty-four had died." At St. Bartholomew's Hospital 135 patients were treated, of whom fourteen have died, nine being brought in dead. The students proved invaluable in carrying patients from conveyances of all descriptions in which they were brought, and a number of men from Smithfield Market came over and rendered great assistance too. It seems to us that the telephone service between France, the coast, and some fixed centre in London might be so organised as to secure that each of the great hospitals should receive at least fifteen minutes' notice in advance of every raid.

### RECUPERATIVE HOSTELS.

WE are glad to have the authority of Sir Frederick Milner, Bart., for the announcement that the Government have now seen their blunder and that in future Sir Frederick has reason to hope that the unfortunate nerve-stricken men to whom he has proved such a friend will be treated in special hostels and not sent to asylums, except in the case of patients who are found to be really insane.

### ESPRIT DE CORPS AT ST. BARTHOLOMEW'S.

THE *Times* of June 9, in a leading article, commented upon the *esprit de corps* which is so characteristic a feature of the large medical schools of the country; Mr. Thomas Hayes, the clerk of St. Bartholomew's, in a letter to the same journal, appearing two days later, pointed out that the idea of utilising this *esprit de corps* in the best interests of the Army Medical Service is no new thing. When the formation of Territorial hospitals was first suggested by Sir Alfred Keogh in 1908, St. Bartholomew's, as the oldest and one of the largest hospitals in the kingdom, was invited to give its adherence to the scheme. The members of the staff, entering at once into the project, were commissioned as officers, whose services would be available on mobilisation, of a complete military base hospital. Within six weeks of the outbreak of the war premises were found at Camberwell, a hospital of 520 beds was equipped, and patients were being received. The nursing was undertaken entirely by past or present members of the nursing staff of St. Bartholomew's, and a majority of the orderlies were students of the hospital. This unit, known as the 1st London (City of London) General Hospital, has since extended its accommodation, and is now equipped for the reception of 1,400 patients. In response to requests from the War Office, the staff of this great Metropolitan hospital has on more than one occasion offered to provide a complete nucleus—medical and nursing—for an overseas hospital.

### ANOTHER LANDMARK AT CARDIFF.

The time has gone by when it was true to say that orthopædics were neglected in this country; but the opening of orthopædic departments which still goes on reminds us how recent has been the recognition of their importance. The latest addition of this kind is the orthopædic section which will be opened in connection with King Edward VII.'s Hospital, Cardiff, on July 5. At the present time eighty civilians or so are attending the section, and when Dr. Fortescue opens it early next month he will be giving formal importance to a new extension of work. This is not the only development of the summer. A ward of fourteen children's cots is about to be opened through the generosity of the Cory family, which is perpetuating the memory of the late Mr. Richard Cory in this way. Moreover, another ward, for twenty children, is also to be opened through the kindness of Mr. Charles Radcliffe. An idea of what this means is given by the fact that these new wards will bring the total number of children's beds in King Edward VII.'s Hospital to 100.

### A REMARKABLE CHANGE AT DEVONPORT.

We have so often alluded to the financial difficulties of the Royal Albert Hospital, Devonport, that it is pleasant to record the new source of strength which it has gained by the voluntary efforts of the staff, of all grades, in the Construction Department of the Dockyard. Mr. W. T. Hockaday, Construction Manager, addressed a War Distress Committee meeting last year, when a representative committee of thirty was created, which has been busy ever since in organising a voluntary collection among the staff. Aided by two successful concerts, the year's work in this direction has led to the sum of £1,000 being handed over to the hospital's funds. The enthusiasm of both men and women has been very gratifying, and the success is only another proof that the way to raise money for hospitals is not by sporadic appeals, though at times these, too, are necessary, but by the creation of a general public interest expressing itself in various organisations of the people in the district which the hospital serves. Such organisations not only provide a bond of good will and an annual revenue, but as it were prepare the soil of public opinion for the issue of special appeals when the need arises for them. The above is the best news we have heard of the Royal Albert Hospital for many a day, and the moral is one which can probably bear an extended application.

### WOMEN ON THEIR OWN.

Another side of the pay-ward movement in this country is seen in the activity of the Professional and Business Women's League. Its object is to provide free treatment in hospitals for those of its members who, through severe illness, have to face the alternative of finding the money to pay the fees of a nursing home, or the hardly less exacting task of entering a general hospital ward. The war, shortly before which the League was established, interrupted its work for a time, but last year it secured the use of a ward in the South London Hospital for Women. From its three hundred members sufficient funds have been obtained to endow private wards in different hospitals, as soon as accommodation can be spared. The honorary secretary of the League is Miss Rawson, and its organiser Mrs. Kineton Parkes. It would be interesting to know what proportion of serious cases occur in this membership, and how far the League is able to calculate an average every year. It is certainly formed at a time when the number of "women on their own" is increasing, and, if well managed, its membership should quickly and largely increase. Hospitals with plans for increasing their pay-wards would do well to keep in touch with its developments. Endowed pay-wards, such as it is seeking to establish, may hasten their development in many institutions.

### THE CONDITIONAL DONATION.

Mr. William Hartmann's offer of £1,000 to the Royal Hospital for Diseases of the Chest, City Road, conditional upon seven other thousands being received before March 31, has attracted to itself four similar promised donations, and, as we predicted, a second and urgent appeal is made to secure other donors to save the situation. It is often incumbent upon the starter of the conditional donation appeal to modify the conditions of the gift, and in this case it is clear that Mr. Hartmann and the four other friends of the hospital (three of them members of the Council) have concurred in extending the period under which the offer holds good. Lord Hollenden, the president and treasurer, in making an appeal for the two extra thousands, wisely does not ignore the possible help of smaller givers, for in these times to give a charitable person the choice between giving £1,000 and nothing is, to say the least, risky. £25,000 in all is asked for. As the whole of the beds of the Royal Hospital are at the present time being used by soldiers, the income and expenditure account, as divided between in- and out-patients, will be an interesting study, as it will show, as clearly as can be shown, if the capitation grant allowed by the Government per bed occupied is or is not sufficient to maintain the bed.

It seems likely, judging from the cost per occupied bed elsewhere, that quite a substantial part of the cost of the in-patient treatment must fall upon the ordinary resources of the institution.

### THE ABUSE OF THE TICKET SYSTEM.

It having been suggested that the out-patient department of the North Herts and South Beds Hospital should be closed, opposition was quickly made, and the result was a compromise in the form of appointing a committee to report. All that the Insurance Act has done in this district appears to be to have reduced the out-patients from 1,000 to 800. The doctors' case is that many are trivial cases, that the ticket system is abused, except in the villages, and that if the times and days of admission could be compressed the already over-worked doctors would be better able to cope with the demands for their services. Dr. Cozens has declared that 50 per cent. of the out-patients had no business there, and Dr. Grellet remarked that the indiscriminate use of letters led to an increase in the drug bill through the consumption of mixtures, though Dr. Gilbertson remarked that there were certain old ladies who liked to come year after year for the same medicine, and would be miserable if they did not get it. Is not the remedy to refer the trivial cases to their panel doctors? The Insurance Act appears to have been almost a dead letter in Hitchin.

### A REAL IMPROVEMENT.

A noteworthy statement was made by Mr. A. Wilson when presiding over the annual meeting of the Birmingham Royal Institution for the Blind. While additional accommodation for workshops is needed, and will be provided as soon as building is permitted once more, the institution has not been able to keep the school full. The reason is that increased care is being taken of infants, so much so that Mr. Wilson declared that of over one thousand children who suffered from ophthalmia neonatorum during the past three years, not one had wholly lost its sight. The work is beginning to tell, and he believed that before long there would not be sufficient blind children to maintain the normal school figure. If this is proved true, it will be a fine example of the value of preventive medicine.

### A JUBILEE FUND STILL UNALLOTTED.

There seems to be a sort of fatality which dogs the heels of funds started in a hurry on the plea of commemorating such public occasions as a sovereign's death or a jubilee reign. The motive is over-exploited as a rule, and while many succeed there are numerous failures. It is the failures which cause all the trouble or linger grievously on, to the worry of all connected with them. Thus a scheme has been submitted by the Charity Commissioners to the Worcestershire County Council for administering the balance of a fund commemorating the Diamond Jubilee of Queen Victoria in 1897. Of the four trustees then appointed two have died, and two more have been proposed. A member protested against the Council being encumbered with a body of important trustees to deal with a paltry sum of money. The income is very small. The chairman of the Council declared that it was worried to death by the Worcester Infirmary, which wanted to grab the greater part of the fund. That is why the Charity Commissioners were applied to. In support of the infirmary, Mr. Bruce Ward declared that it was a scandal that the money had been locked up for so long. The chairman then explained that the infirmary asked for the money to be allotted in relation to the number of beds then in existence, but since 1897 two wards had been closed. The infirmary, therefore, claimed more than its fair share. Again we have to notice the trouble which invariably follows on the foolish and faint-hearted policy of closing wards. The infirmary, as this shows, has forfeited public confidence in this respect. It was bound to do so. Trustees are to be appointed.

### MEAT INSPECTION PROBLEMS.

The Royal Sanitary Institute will hold a meeting at the Town Hall of Weston-super-Mare on Saturday, June 30, at 11.30 a.m., when a discussion will be held on meat-inspection problems. The discussion will be opened by a paper by Dr. W. G. Savage, M.D., B.Sc. (Medical Officer of Health, Somerset County Council), on "Meat Inspection in Rural Districts," to be followed by a paper by Mr. G. M. McGregor (Meat Inspector of Cardiff) on "Cold Storage and Meat Inspection." Visits will be made to the Weston-super-Mare new abattoirs, and a demonstration of diseased meat and animal parasites will be arranged in the County Laboratory.

### ULSTERMAN'S BEQUESTS TO CHARITIES.

No fewer than twenty-eight specific bequests to charity are made in the will of the late Mr. James Bruce, of Belfast, a director of Messrs. Dunville and Co., Ltd., distillers, and a prominent hunting and driving man. Mr. Bruce, who died at the age of fifty-two, left personal estate valued at £681,000. The largest of the charitable bequests is made to the Thompson Memorial Institute, Lisburn, which receives £7,000; £1,000 each are given to the Belfast Royal Hospital, the Society for Providing Nurses for the Sick Poor, Belfast, and the Society for the Relief of Distressed Irish Ladies. A number of other charities were left £500 each, and these include the Maternity Home, the Hospital for Sick Children, and the Consumption Hospital, all in Belfast. Mr. Bruce was evidently no religious bigot, for Roman Catholic and Protestant institutions alike benefit by the thoughtful provisions of his remarkable will; sums also are left for distribution amongst the poor to ministers of several religious denominations, and hardly any branch of charitable enterprise, whether for the sick, the deaf, the dumb, the blind, or the poor, appears to have been overlooked. In the failure of issue to the testator's brothers other bequests will be available for charitable purposes. Mr. James Bruce evidently gave a great deal of thought to the preparation of his will, very properly devoting the most of what he had to give away to the district where he lived so long and where his business was conducted.

### BEQUEST FOR A NEW HOSPITAL.

Large bequests to charity have been frequent of late, and, while most testators are content to leave unconditional gifts to established institutions, occasionally provision is made for the starting of a new charity or for the association of the name of the person who has left a legacy with a bed, a ward, or a wing. Lieutenant-Colonel Sir John Howard, of Brighton, a director of the North British Railway Company, and designer and contractor of the Palace Pier at Brighton, left estate to the gross value of £284,424. The largest specific bequest is £40,000 in trust to establish a "John Howard" Hospital, primarily for surgical cases, and for the use of patients able and willing to pay reasonable and ordinary fees, or to assist any existing hospital or for other hospital purposes in Brighton. Another interesting bequest in this notable will is £33,600 and land on which it is built for the "John Howard" Convalescent Home, and for the erection of 24 cottages for the use and lodging of trained hospital nurses whose health has broken down, and for the payment of ten shillings a week to each of the inmates. Sir John Howard, in addition, provided handsomely for other Brighton charities, and left the residue of his estate to be distributed by his executors, at their discretion, in Brighton and other parts of England.

### LOTTERIES FOR CHARITY.

In referring to the lottery scheme promoted by the Glasgow Tramway Corporation in our issue of May 19, page 124, we expressed some surprise that a money lottery pure and simple was passing unchallenged. We now learn that the Crown authorities have warned the Glasgow Corporation that proceedings will be taken if the system is continued. Lotteries for charity are much in the air at the present time; only a few days ago the newspapers announced that a boy had won a prize of several thousand pounds as the result of a sweepstake organised for Red Cross funds at the Smithfield Meat Market. Indeed, the lottery has taken many forms for charity since the great war began. Apparently where the prize is not in the form of money a licence can be obtained from the Board of Trade to hold a lottery, and a movement of this kind, in which the prizes are paintings and drawings by well-known artists, is being organised on behalf of St. Dunstan's Hostel for Blinded Sailors and Soldiers. There can be no harm in such a lottery as that for St. Dunstan's Hostel. It has already caused many people to render excellent personal service, and we wish it a triumphant success.

### THE "FIFTH."

A proposed land scheme for the benefit of wounded soldiers is the principal subject treated in the latest issue of the magazine of the 5th London General Hospital (St. Thomas's). If 100 acres of land could be obtained as a gift from some landowner, it is planned to place on it twelve four-roomed cottages, to be occupied by twelve convalescent soldiers. The main estate would be divided into twelve parts, each of which would be devoted to the cultivation of a separate crop: sugar beet, strawberries, potatoes, lettuce, mustard and cress, are mentioned. It is believed that expert guidance and tools would be given for the sake of the advertisement, and that a couple of cart-horses could be obtained from the Army. If successful, the scheme, of course, could be developed. To make it so, criticism would at once suggest that the proposal for devoting each parcel of land to a separate crop be modified so as to have the ground in continuous use throughout the year. This, in fact, is so obvious that we are surprised that it did not occur to the proposer, M. W. H. The magazine contains two photographs of English prisoners of war in a Thuringian hospital. There is also an elaborately illustrated "massage alphabet." There is a concluding "List of Patients," which is a useful characteristic, and one that may be valuable for reference in times to come.

### STATE MILK.

Lord Rhondda has created a precedent for the State feeding of children. He has given his consent to a contribution by the Marylebone Borough Council of £50 towards a local fund which is being raised by the Mayor for the supply of milk to delicate children. Of course, some Boroughs have depôts for the sale of sterilised milk, but this is the first time they have been empowered to make a direct contribution from the rates for supplying food to delicate children. The Local Government Board is particularly anxious regarding child life, and has suggested that Marylebone should arrange for additional visits to be made by nurses of the District Association to cases of measles occurring among very young children in selected areas where conditions are unfavourable to health. The Council, however, thinks it better to deal only with really bad cases.

### THIS WEEK'S DRUG MARKET.

The upward movement in the values of salicylic acid and sodium salicylate continues owing to the scarcity of supplies; anything offering is quietly picked up. Only small parcels of phenacetin are available, and the high price is well maintained. The price of resorcin has receded somewhat, and the drug is being produced in this country. Only trifling quantities of atropine sulphate are available, and the value has an upward tendency. English refiners of camphor have raised their quotations. Quinine is quiet, but the price is well maintained. A further rise in the price of hypophosphites is probable as makers are very busy and behind in deliveries. Owing to the increased cost of materials a further rise in the value of glycerophosphates is probable; makers are using imported glycerine, which is obtainable only at high rates. The English henbane crop is now being harvested and the yield is about the average. Growers of belladonna report that the plants look about the average. Lavender plants look fairly good, and given dry and warm weather there should be a fair yield.

